# Supplementary material for: Staff perspectives on the impact of COVID 19 on the delivery of specialist domestic abuse services in the UK: A qualitative study
Source: PLOS Glob Public Health. 2022 May 5;2(5):e0000310. doi: 10.1371/journal.pgph.0000310 (PMC10021249; doi:10.1371/journal.pgph.0000310)
Supplement: S1 Text — (DOCX) [file pgph.0000310.s001.docx]

**Interview Topic Guide**

**Introduction**

- Thank you for seeing me today and offering to take part in this study
- I would like first to outline the study so that you are able to decide whether you wish to continue (recap participant information sheet)
- I also want to remind you that all of the information you share will be confidential and you have the right to withdraw from the study at any point
- I have received your written consent but want to check at this point that you are still are happy to proceed?
- I have a number of questions that relate to your recent working experiences that I would like to ask
- Feel free to ask questions at any stage during the interview

**Topics/Questions**

**Background Info**

1. How old are you
2. What is your preferred gender?
3. How long have you worked for HSS and what is your current role?

- I am going to ask questions that relate to each stage of support that you offer victims of domestic abuse, I have categorised these questions into Initial Contact, Assessment, Intervention and General information

**Initial Contact**

- The first questions I will ask relate to service delivery before 23^rd^ March 2020

1. How would victims usually contact the service for support e.g., telephone/online?
2. Were there any times that were busier than others, for example during office hours or evening or weekends?
3. Would there be any initial safety checks carried out at this point; if so, what would have been?

- **The next set of questions relate to initial contacts made to the service by victims between 23^rd^ March – July 2020**

1. Tell me about how the service and the support you offer has changed because of COVID-19
2. During this time what different methods of contact were made available to victims seeking support?
3. How did victims actually contact the service during this time?
4. Did the method that victims used to contact the service change during this time period, if so how? Why do you think this was?
5. Where there any times that were busier than others – did this impact on waiting lists?
6. Did your safety checks change or were there different approaches taken to ensure that people were safe/at risk?
7. Has anything else changed?
8. Have the new processes impacted on engagement of victims?
9. What’s working well, do you have an example of good practice you would like to share?
10. Is there anything that you are worried about regarding the new adaptations?
11. Are there new methods that you would like to see a permanent feature in your delivery model and why?

**Assessment**

- The next set of questions relate to the assessment process and/or any risk assessment you might carry out at each contact
- The first few questions relate to services offered before 23^rd^ March 2020

1. Following initial contact, how would you carry out the required assessment process – what did this look like?
2. What risk assessments and/or tools did you use?
3. How did you safety plan?
4. What types of abuse were most common?

- The next questions relate to services offered between 23^rd^ March – 6^th^ July 2020

1. Following initial contact, how were assessments carried out – what has changed?
2. Have waiting lists been affected?
3. How have you been carrying out risk assessments – what tools have you used, what’s changed about them?
4. Are there any additional questions/safety checks carried out?
5. How have safety plans changed?
6. Has the type of abuse present changed, if so, how?
7. How have the levels of risk within families changed e.g., are there more high level?
8. Have the new methods impacted on engagement?
9. Did this impact on the intervention offered?
10. What has worked well during this time period, is there an example of good practice you would like to share?
11. What are you worried about in relation to the adaptations that have been made can you give me an example?
12. What new methods would you like to see a permanent feature in your delivery model and why

**Intervention**

- The next set of questions relate to the delivery of specialist interventions

1. Can you describe what interventions were offered and how they were delivered to an adult victim before 23^rd^ March 2020?
2. What were the levels of engagement like before 23^rd^ March, were there any issues with waiting lists?
3. How did the intervention change from the 23^rd^ March onwards?
4. How have levels of engagement from victims changed over this time e.g., more/less likely to engage in set appointments virtually?
5. How have waiting lists been affected?
6. Have you adapted any tools, if so, how were they changed?
7. What has worked well over this time?
8. Is there anything that you have been worried about in relation to the adaptations that have been made?
9. What new methods would you like to see a permanent feature in your delivery model and why

**General**

- The next set of questions relate to more general observations you might have made about your practice between 23^rd^ March – July 2020

1. What inequalities have you observed/identified or additional challenges faced by victims that have been made worse because of the adaptations e.g., access to online?
2. How do you manage non-verbal cues when services are delivered online If e.g., body language as you might do in face-to-face contact, if so, how are you mitigating against this?
3. What sort of specialist training have you had to enable you to continue to deliver services?
4. Have the new methods of delivery had any other unintended impact on you as a worker, how have you felt during this time?
5. Is there anything you would like to highlight that you feel we haven’t had the opportunity to discuss today?
6. What areas would benefit from further study?

End of interview – thank you for your participation
