# Supplementary material for: Staff perspectives on the impact of COVID 19 on the delivery of specialist domestic abuse services in the UK: A qualitative study
Source: PLOS Glob Public Health. 2022 May 5;2(5):e0000310. doi: 10.1371/journal.pgph.0000310 (PMC10021249; doi:10.1371/journal.pgph.0000310)
Supplement: Attachment — Submitted filename: PLOS GLOBAL PUBLIC HEALTH response to reviewers.docx [file pgph.0000310.s002.docx]

Response to Editors Comments:

**Reviewer 1 has made a suggestion to condense the manuscript. Please review the comment and see if there are areas you would like to condense. However, PLOS Global Health** **does not have a word limit, and so you are not required to reduce the text significantly if you do not feel you can do so while still adequately conveying the necessary components of the manuscript.**

We have reviewed the manuscript for areas where we could condense following the suggestion from reviewer 1 however as we had already condensed our data significantly in order to draft the original manuscript, we do not feel there is room for further significant condensing while also still adequately conveying the necessary components of the manuscript.

**Reviewer 2 has suggested that "Every paragraph in discussion should be linked to something you found and want to discuss relative to external literature". Please consider how you can better tie in your discussion to the existing literature, but not every paragraph needs to do so as suggested.**

We have significantly restructured the discussion and conclusion sections. We have made sure that external literature is explicitly tied into our findings reminding readers of what “we found”.

**Please update the completed 'Competing Interests' statement, including any COIs declared by your co-authors. If you have no competing interests to declare, please state "The authors have declared that no competing interests exist". Otherwise please declare all competing interests beginning with the statement "I have read the journal's policy and the authors of this manuscript have the following competing interests:"**

The competing interests statement has been updated as requested.

**We notice that your supplementary information is included in the manuscript file. Please remove them and upload them with the file type 'Supporting Information'. Please ensure that all Supporting Information files are included correctly and that each one has a legend listed in the manuscript after the references list.**

We have removed the interview topic guide from the manuscript file and uploaded it as the file type ‘supporting information’. A legend is listed in the manuscript after the reference list.

**We have noticed that you have uploaded supporting information but you have not included a list of legends. Please add a full list of legends for all supporting information files (including figures, table and data files) after the references list.**

A legend for the supporting information file has been added to the manuscript.

**Please amend your Data Availability Statement and indicate where the data may be found.**

We have updated the data availability statement to read: “We have made excerpts of the transcripts relevant to the study available within the paper however there are ethical restrictions on sharing the de-identified data set further. The data contain potentially identifying and sensitive participant information and we do not have participant consent to share this dataset. Data requests may be sent to Northumbria University Research Ethics Committee (ref 24857) (nick.neave@northumbria.ac.uk).”

Response to Reviewers'

**Reviewer #1: Thank you for the opportunity to read and review this paper, on the topic of UK domestic abuse services in the context of the covid-19 pandemic. This is an issue of obvious public health relevance, and this study can provide a relevant contribution. Provided that some revisions are made, as pointed to below, I would recommend this study for publication.**

Thank you for your interest in our manuscript and for your helpful comments. We have revised the manuscript accordingly and hope you now find it suitable for publication.

**I think the discussion should delve a little bit more into what insights can be generalized and carried into a ‘back-to-normal’ versus ‘staying-online’ state of affairs. In the limitations section, the authors do state that this study is made in a particular context and that the results may therefore not be representative for other services/places, but I think this issue should be discussed a little bit more since domestic abuse services in many countries across the world have faced similar challenges. If nothing else, the authors could make a claim that findings and themes are likely relevant beyond the specific setting, and that similar research in other contexts is warranted. Also, while we don’t know at this point when and to what extent services will go ‘back to normal’, the authors could discuss a bit more clearly which adapted practices should be kept in place or developed and which should revert back to face-to-face praxis, if the latter is possible, or what should be kept in mind should services need to be kept, or temporarily return to, online-only.**

We have added to the limitations section that since domestic abuse services in many countries across the world have faced similar challenges our findings are likely to be relevant beyond this specific setting and similar research in other contexts is warranted. We have also discussed more clearly which adapted practices should be kept in place or developed and which should revert back to face-to-face in the future.

**The manuscript could be condensed. At the moment it’s quite long, and while it is helpful to get this much detail and this many quotes, I think the readability would improve if some quotes (which don’t add substantially to what is already mentioned in the text) were omitted and the text revised with abbreviation in mind. I think Table 1 is superfluous, as it doesn’t provide information that isn’t or can’t be stated in the text (i.e., the age of the participants was between 20 and 55 years, and half had 1-4 yrs of experience while the other half had 5 yrs or more).**

Thank you for this comment. We have reviewed the manuscript for areas where we could condense however as we had already condensed our data significantly in order to draft the original manuscript, we do not feel there is room for further significant condensing while also still adequately conveying the necessary components of the manuscript along with the supporting data. As the editor has noted, PLOS Global Public Health does not have a word limit and so we are not required to reduce the text significantly. In addition, we think table 1 is necessary because, while we agree some of the information could be summarised in the text, it allows the reader to identify anonymised characteristics of individual participants whose quotes are presented.

**Along related lines, the manuscript would benefit from being proof-read throughout. Many sentences are very long (e.g., line 274-278, 299-304), some are a bit unclear** **(e.g., p 4 “Learning from studies… has shown”; p 10 “Participants reflected that this was due to the nature…” and “Both participants described calls…”; p 167 “All participants commented on…”) and others are not complete (e.g., sentence starting on line 99). The paragraph starting on line 64 is long, and should be broken up into two. While there are different approaches to quotation,** **I would recommend slightly editing some of the quotes for punctuation and syntax (e.g, line 244-247, 269-272).**

Thank you for identifying these errors, the manuscript has now been thoroughly proof-read. In particular we have revised long sentences (e.g., line 274-278, 299-304 on original manuscript) clarified some unclear sentences (e.g., p 4 “Learning from studies… has shown”; p 10 “Participants reflected that this was due to the nature…” and “Both participants described calls…”; p 167 “All participants commented on…”) and completed others (e.g., sentence starting on line 99). The paragraph starting on line 64 has been broken up into two. We have edited some of the quotes for punctuation and syntax (e.g. line 244-247, 269-272).

**Regarding the decision on the number of interviews, I think the reference “based on previous work” is a bit vague. This is perhaps particularly so as the authors refer to piloting throughout the first interviews and making amendments accordingly. Did the authors find that they reached a level of saturation in the material? Also, with regards to sampling via author e-mails, the authors state that their “approach was used as there were existing work-based relationship with two of the potential participants and the author (HR) may have been known to other participants”. This is a bit unclear to me. This method was chosen above what alternative?**

The decision regarding number of interviews was based on our previous research experience – this has been clarified within the text and a citation added. Saturation is now a somewhat contested term hence the discussion of data sufficiency within the methods section however we have also acknowledged a potential lack of saturation as a limitation. “This approach” referred to the way that the author and interviewer introduced herself to potential participants (as a student researcher rather than a commissioner). This has been clarified within the text.

**I think it would be helpful if the authors elaborate a little bit, before or in the beginning of the results section, about the changes in practices made due to the lockdown, including the special measures that were introduced. Now such measures are mentioned here and there the results (Rail to Refuge, COVID-19 Secure, remote granting of non-molestation orders, workforce development package/additional emotional health and wellbeing support). While this is all good, I think an overview of changes and adaptations – including ones that perhaps aren’t mentioned in the paper at this point - would be useful.**

Thank you for this helpful comment. We have added a section to the methods section entitled “context” in order to describe the changes in practices made due to the lockdown restrictions.

**On p 6, it would be interesting to find out early on (although it is mentioned later that it is voluntary) if the service provider was voluntary or part of a state-funded social service. Some more information about it may also be interesting: how many people it employs/involves, how many clients it serves...** **Also, please provide a reference to the COREQ checklist.**

Thank you for this helpful comment. We have added a section to the methods section entitled “context” in order to provide more information about the service. A reference to the COREQ checklist has also been added.

**On p 8, regarding the analysis, the authors state that “The themes identified were re-analysed so that they became more refined and relevant and were reviewed by both authors on an ongoing basis”. Could this be a specified slightly – what does “on an ongoing basis” mean?**

This sentence has been made more specific and now reads: “The themes identified were re-analysed so that they became more refined and relevant and were reviewed by both authors at regular monthly supervision meetings.”

**On p 2, “connections with socio-economic disadvantage and social welfare” is a bit vague - can this be specified somewhat?**

This sentence has been made more specific and now reads: “Domestic abuse is a significant public health issue, present within all social classes however there is recognition that low income, economic strain, and benefit receipt is associated with heightened vulnerability to domestic abuse.”

**A few times, the word “business” is used for the service provider activity. Is this an appropriate concept to use?**

Thank you for identifying this. The most appropriate concept to use is service. Any reference to “business” has now been amended to “service”.

**On line 205, does “make ready refuge rooms” mean prepare rooms or make new ones?**

“Make ready refuge rooms” refers to preparing the room rather than making new ones. This has been clarified within the text.

**Is “recidivism” a better term to use than “repeat”?**

Thank you for this suggestion. We have used the term recidivism rather than repeat when referring to perpetrators reoffending.

**On p 3, “ending March” – does this mean ending in March, or ending on March 1st/31st?**

This refers to year ending 31^st^ March 2019. We have clarified this within the text.

**On p 5, the very specific time frame doesn’t seem to rhyme with the qualitative approach used in the paper, unless there is a particular reason for it.**

The specific timeframe refers to the period of lockdown within the UK as stated in the manuscript.

**Finally, in the introduction the authors list a few aspects of how lockdown may affect domestic abuse. A suggestion, although this is up to the authors, is that the relevance of social isolation as a correlate to [1] and as central to the dynamic of [e.g., 2] (forms of) domestic abuse is also mentioned.**

**[1] Lanier C, Maume MO. Intimate Partner Violence and Social Isolation Across the Rural/Urban Divide. Violence Against Women. 2009;15(11):1311-1330. doi:10.1177/1077801209346711 Social isolation correlate**

**[2] Leone, J. M., Johnson, M. P., & Cohan, C. L. (2007). Victim help seeking: Differences between intimate terrorism and situational couple violence. Family relations, 56(5), 427-439.**

Thank you for this suggestion we have added the relevance of social isolation to domestic abuse citing the suggested references.

**Reviewer #2: This paper provides important information about the context of DV service provision in the Covid-19 pandemic and can be useful for other organizations that are similarly adapting their work. The paper would benefit from some edits, as noted below, and in particular the discussion needs to be rewritten and description of a theoretical framework or foundation should be tied into the methods section. While generally well-written, copyediting is needed to rectify some commas in the wrong place throughout the paper and to remedy overuse of the passive voice, especially in the methods section. I look forward to seeing a future version of this important paper, which I hope will be published after appropriate revisions and will benefit the field.**

Thank you for your interest in our manuscript and for your helpful comments. We have revised the manuscript accordingly and hope you now find it suitable for publication.

**The lack of data availability is suitable for the field of domestic violence, though it would be useful to state that "data are available upon reasonable request".**

We have updated the data availability statement to read: “We have made excerpts of the transcripts relevant to the study available within the paper however there are ethical restrictions on sharing the de-identified data set further. The data contain potentially identifying and sensitive participant information and we do not have participant consent to share this dataset. Data requests may be sent to Northumbria University Research Ethics Committee (ref 24857) (nick.neave@northumbria.ac.uk).”

**Abstract
• A growing number of qualitative researchers, notabely Braun and Clarke who wrote one of the most cited guides to thematic analysis, and who you cite (citation 17) argue that themes can’t “emerge” – suggest you update this language (in abstract and results section).**

Thank you for this comment. We agree that this is now contested terminology and have revised the language in both the abstract and results sections to state that themes were developed from the data.

**Introduction
• You talk about the 24% increase from 2018 to 2019 in violence – before the pandemic. Why is that increase relevant, and do you have ideas to share or evidence on why that is the case? Could the further increase once Covid began not be in part a continuation of that trend? Not sure why the 24% increase figure is relevant to your narrative.**

Thank you for highlighting this, in hindsight we do not think reporting the increase in domestic abuse related crimes from 2018 to 2019 is relevant. We have therefore deleted this sentence.

**• page 4 row 74- “Learning from” is not needed - start with “Studies show”**

This sentence has been amended and as such “learning from” has been removed.

**Methods
• You talk about this as an evaluation study (filling the gap in terms of no evaluations) but the study design does not mentioned evaluation or what type of evaluation design you use.**

Thank you for this comment. Our study examined the impact of the COVID 19 pandemic on domestic abuse services rather than being a formal evaluation therefore references to evaluation have been removed from the manuscript.

**• p 6 study design – not a full sentence- no verb and also too long of a sentence. Be direct “We carried out”.**

This sentence has been amended and now includes the phrase “we carried out”. It has also been reduced in length.

**• Could you delete “firmly within the boundaries of” - Not sure what that means in the context of the sentence. Also you don’t cite or define phenomenology**

We have deleted “firmly within the boundaries of” and have revised this sentence to include a definition and citation.

**• SAMPLE: in March and July 2020 (did you mean “between”? What about April, may, June)**

Yes, we meant between March and July 2020. This has been clarified within the text.

**• It is unclear what you mean by p 7 row 131 “This approach was used…” – please clarify what you mean by this sentence- what approach, why did that help the situation. what does it mean to be known to other participants and how does that influence things and your approach. Be specific.**

“This approach” referred to the way that the author and interviewer introduced herself to potential participants (as a student researcher rather than a commissioner). This has been clarified within the text.

**• Interview guide development – how did you come up with the 6 areas? Does it draw on literature – if so cite. Or on past studies? This relates to the next point.**

The interview topic guide was developed through discussion between the authors. This has bee clarified within the text.

**• Not theoretical framework underpinning the study is mentioned. Please describe this- beyond experience in practice from the authors work, what theory or literature has shaped or influenced the design of the study**

As stated in the manuscript we adopted an interpretive framework with the purpose of establishing the shared essence of a phenomenon through lived experience. This framework guided the choice of method for data collection (interviews) and analysis (thematic). We have clarified this further within the manuscript.

**Use of passive voice
• Passive voice is used throughout, even in sentences that talk about the first author as a person. Therefore, please adjust throughout the paper to own the work you do and use active voice. Some examples you could change are “key findings are presented”**

Although use of the passive voice is common in academic writing, we have adjusted the manuscript with use of the active voice. While changes have been made throughout the manuscript, we have also revised the example given here.

**• RECRUITMENT: “were used” – change to direct tense. You are already saying one of the authors was involved in recruitment so please reduce use of indirect voice/language. Also, “were emailed by”**

“were used” has been changed to “she used” and “were emailed by” has been changed to “HR emailed potential participants”.

**• Data analysis: “was adopted” – can this be direct form not indirect**

“was adopted” has been changed to “we adopted”.

**Results
• Row 160 “However this simply reflected” can be changed to ‘reflecting’ - however is not relevant there.** **Also, is this typical of the gender breakdown in the domestic violence space? if so can note that this represents the work force.**

“however this simply reflected” has been changed to “reflecting”. As this is typical in domestic abuse service this point has been added to the manuscript.

**• In the table the years of work is 1-4, 5-10, 10+ = unequal split (4 years, 6 years). What about 1-5, 6-10, 10+?**

Thank you for this suggestion. The suggested groupings work and the paper has been amended accordingly.

**• Page 11 row 201 – what does “out of the local area” mean – used in a few places but we don’t know what you are referring to.**

This refers to areas outside of the north-east of England. This has been clarified within the manuscript.

**• page 16 “being truthful” – in the quote obviously it should stay to be true to the original meaning, but in your text perhaps change the wording to explain what that means. Currently sounds like they don’t trust the client; whereas in reality it is that sometimes people feel uncomfortable, ashamed or unsafe to disclose the extent of their abuse or their situation.**

Thank you for this comment the text of the manuscript has been changed to reflect that this statement is not about trusting whether the client is honest or not but about whether they are able to disclose the extent of their abuse.

**• Structure of results section: Each theme title (quote) represents just part of a theme. Because it gives an incomplete picture, I recommend adding a topic sentence or paragraph to top of each theme to let the reader know what to expect.**

We have added a topic heading to each theme title to give the reader more information about each theme.

**• Theme 4: in the opening paragraph when talking about “biggest impact” can you say in what direction or the type of impact? if you say it up top the reader will know more of what is coming – easier for the reader**

Thank you for this suggestion. We have added this opening sentence to read “biggest positive impact”.

**Discussion
• The discussion needs restructuring. Some paragraphs of the discussion only mention external literature and don’t explicitly tie in to your findings. Every paragraph in discussion should be linked to something you found and want to discuss relative to external literature – please make sure this is the case as it will strengthen your discussion. Each paragraph should have a topic sentence that tells you what the paragraph is about – linked to something you found and discussed in the results. It should also remind us that you found it – “We found” or “our study demonstrates/ higlights” – make it easy for the reader to see how what you discussed in the results shows up again in the discussion, and make it clear what the take away is – help the reader interpret your results in the context of external literature. The reader won’t always remember what you found vs. what the external literature says so be explicit about when it is your own finding vs what others have said.**

Thank you for these helpful suggestions. We have significantly restructured the discussion and conclusion sections. We have made sure that external literature is explicitly tied into our findings reminding readers of what “we found”.

**• page 30 last sentence: “This study has highlighted” – why are there two citations if you are actually talking about the findings of your own study?**

Thank you for highlighting this. We have clarified our study findings and those to which we have cited.

**• In the opening paragraph of the discussion, and again in the conclusions, please remind the reader the context/location “In this UK-based study of XXX, we found that…” or something like that.**

Thank you for this suggestion we have reminded the reader of the location and context of the study in the discussion and conclusion sections of the manuscript by adding “In this UK-based study of domestic abuse services delivered during the COVID 19 pandemic”.

**• p 32 row 709 – “despite these positives” – this seems unrelated to the men’s care content that is just before it. . could be better as start of a new paragraph**

We have followed this suggestion and started a new paragraph at this point however we have also deleted the phrase “Despite these positives”.

**Limitations
• how might it have influenced it?**

We assume that you are referring to the existing working relationship here? Therefore we have added to the manuscript that this may have resulted in socially desirable responding or the tendency of respondents to reply in a manner that would be viewed favourably by others.

**• all female workforce- worth mentioning that it is typical in that field so the sample is broadly representative of the workforce**

We have added that a predominantly female workforce is typical of domestic abuse service and so the sample is broadly representative to the limitations section.

**Conclusion
• Opening sentence – specify the field – domestic violence services. and add that you found it “our study shows” for example. Otherwise it’s a generic statement.**

We have added the field of study to the opening sentence of the conclusion and have stated that we found…

**• I think first two paragraphs of conclusion would be better placed at top of the discussion section as they would give a overarching overview of what you found.**

Thank you for this helpful suggestion we have moved the first two paragraphs of the conclusion to the top of the discussion section.
