# Supplementary material for: Staff perspectives on the impact of COVID 19 on the delivery of specialist domestic abuse services in the UK: A qualitative study
Source: PLOS Glob Public Health. 2022 May 5;2(5):e0000310. doi: 10.1371/journal.pgph.0000310 (PMC10021249; doi:10.1371/journal.pgph.0000310)
Supplement: Attachment — Submitted filename: PLOS GLOBAL PUBLIC HEALTH response to reviewers 2.docx [file pgph.0000310.s003.docx]

**Journal Requirements: Please review your reference list to ensure that it is complete and correct. If you have cited papers that have been retracted, please include the rationale for doing so in the manuscript text, or remove these references and replace them with relevant current references. Any changes to the reference list should be mentioned in the rebuttal letter that accompanies your revised manuscript. If you need to cite a retracted article, indicate the article’s retracted status in the References list and also include a citation and full reference for the retraction notice**

We have reviewed our reference list to ensure it is complete and correct. We have not cited any retracted papers or made changes to the reference list beyond adding a missing publication date.

**Thank you for sharing the revised draft of your paper. This version is improved and ready for publication pending a few minor revisions. Please find here the particular details to be addressed.**

Thank you. We are pleased that you believe the manuscript has been improved and is ready for publication pending the minor revisions with we have now addressed (see below).

**On page 22 you first bring up the inspire programme. This might be better described briefly in the new Context section you have added, as this is a part of the organisation's work. Then, in Results you can discuss the change in how Inspire was implemented without having to first introduce the programme. The description seems better suited for the context/methods section where you introduce other programs like the Rail to Refuge scheme.**

Thank you for highlighting this. We agree that moving the description of the Inspire Programme to the new context section makes much more sense. To this end we have moved the description of the Inspire Programme into the methods section and out of the results.

**I think your revision of the discussion section reads better now. Upon reading the new draft, it occurred to me that those first two paragraphs [moved up from the conclusion of your prior draft] now include both a high levels summary of your findings and also some details about implications of those findings. I suggest you consider cutting the implications out and moving each set of implications down into a paragraph where that topic is discussed in more depth. Specifically, I'm referring to lines 696 (starting at "Remote working") through 704, and lines 711 (starting at "Client safety" through 716 (ending at "and/or intervention") and lines 717 (starting at "Services should not" through 719]. For example, the implications about Client safety (lines 711-716) could be moved to paragraph 4 about that topic. Similarly with the other implications, they may fit better in other paragraphs.**

We are pleased that you feel the discussion section now reads better however in line with your suggestions we have moved the implications paragraphs to a paragraph where the topic is discussed in more depth.

**In the conclusion on line 805 you say "clear evidence of learning". I don't quite understand what this means. I think you mean your findings can be applicable to development of future services? Please adjust wording to clarify.**

Apologies if the phrase “clear evidence of learning” was not clear. We have adjusted the wording to clarify that “we found clear evidence that can be applied to the development of future specialist service delivery models”.

**On line 811 you take about the Inspire Programme, which limits your findings to that programme. Would it be suitable to say “or other similar empowerment programmes” or “empowerment programmes such as the inspire programme” to make the point that empowerment programmes are a useful addition and should be contemplated in future service design, and adapted/implemented in the ways that make sense in each context?**

Thank you for this helpful suggestion. We have amended the sentence to read “…empowerment programmes such as the Inspire Programme…”

**On lines 815-817 you speak about the Rail to Refuge and Non-molestation orders and say they should be retained. Please add a few words about WHY they should be retained? Consider if there is a way to make this comment a bit more relevant beyond the particular programme or order (as with the Inspire programme comment above) - that is, this programme does something that is useful, but maybe other programmes or initiatives could do that too- so what is it about those programmes that needs to be maintained? Wording it this way can make your findings a bit more applicable elsewhere.**

We have amended lines 815-817 in line with your helpful comments. This section now reads: “We would also encourage the retention or implementation of similar national schemes to the Rail to Refuge Scheme as it removed pressure on both victims and services by allowing victims of domestic abuse to travel free of charge to be able to access emergency accommodation anywhere in the country. Retaining remote hearings for granting of injunctions such as Non-molestation Orders would reduce stress and wait time for victims.”
